# Delivery of Anti-VEGFA Antibody by Plant-Derived Exosome-like Nanovesicles Alleviates Corneal Angiogenesis and Fibrosis via Topical Ocular Administration

**DOI:** 10.3390/molecules31173026

**Published:** 2026-08-28

**Authors:** Xuan Chen, Qian Li, Hong-Ping Cui

**Affiliations:** 1 School of Medicine, Tongji University, Shanghai 200092, China; 2311241@tongji.edu.cn (X.C.); liliqq@tongji.edu.cn (Q.L.); 2 Department of Ophthalmology, Shanghai East Hospital, School of Medicine, Tongji University, Shanghai 200120, China

**Keywords:** plant-derived nanovesicles, anti-VEGFA antibody, ophthalmic hydrogel, corneal neovascularization, corneal fibrosis, drug delivery, corneal alkali burn

## Abstract

**Background:** Pathological angiogenesis and stromal fibrosis after ocular chemical injury are primary drivers of corneal opacification and permanent vision loss. Topical administration of anti-VEGFA antibody is hampered by rapid tear clearance and poor penetration across the corneal epithelial barrier, resulting in insufficient intraocular drug accumulation and suboptimal therapeutic efficacy. Although intraocular injection elevates local drug concentration, this invasive procedure carries risks of complications and is not suitable for long-term repeated treatment. This study constructed red cabbage-derived exosome-like nanovesicles (Rabexo) to load an anti-VEGFA antibody, formulated into PVA/HA hydrogel, aiming to develop a topical sustained-release ocular delivery system for alleviating corneal angiogenic and fibrotic lesions. **Methods:** Rabexo was isolated and physicochemically characterized. Anti-VEGFA antibody was encapsulated into Rabexo via electroporation to prepare aV-Rabexo, which was further incorporated into commercial PVA/HA hydrogel. Cellular uptake, anti-angiogenic functions were verified in vitro, and therapeutic efficacy was evaluated in a mouse corneal alkali burn model via ophthalmic topical administration. **Results:** The prepared Rabexo showed uniform size and favorable colloidal stability with 40.3% antibody encapsulation efficiency. aV-Rabexo exhibited stronger in vitro anti-migration, anti-proliferation and anti-tube formation effects than free antibody. In vivo, aV-Rabexo-Gel substantially suppressed corneal neovascular area and length, restored stromal collagen arrangement, and downregulated CD31, VEGFA and α-SMA expression at day 14 post-injury, with statistically significant differences compared with the free antibody gel. **Conclusions:** This plant-derived nanocarrier hydrogel system realizes synergistic therapeutic effects via nanocarrier delivery, antibody neutralization and prolonged ocular retention. With advantages of easy production and low immunogenicity, it provides a promising non-surgical strategy for treating ocular surface pathological angiogenesis and fibrosis.

## 1. Introduction

The cornea maintains a physiological avascular and transparent microenvironment to guarantee normal visual function. Severe ocular insults, including chemical burns, mechanical trauma and chronic inflammation, consistently trigger aberrant VEGFA upregulation, which initiates pathological corneal angiogenesis [1,2]. Progressive neovascularization further exacerbates local inflammatory responses and activates fibroblast-myofibroblast transition, ultimately leading to stromal collagen disorder, irreversible fibrosis and corneal scarring. The combined pathological progression of angiogenesis and fibrosis constitutes the primary cause of poor visual prognosis after severe corneal injury, yet effective topical intervention strategies remain limited in clinical practice [3,4].

Anti-VEGFA monoclonal antibodies have been widely adopted to inhibit pathological ocular angiogenesis [5,6]. However, conventional topical eye drop administration faces two core delivery obstacles. The rapid turnover of tear fluid eliminates most surface-applied drugs within several minutes, while the tight junction structure of the corneal epithelium severely blocks the transmembrane penetration of macromolecular antibodies, resulting in extremely low ocular bioavailability [7,8]. Frequent high-dose medication is required to compensate for insufficient drug accumulation, which increases the risk of epithelial irritation and reduces patient compliance. Invasive injection therapies, including subconjunctival and intrastromal injections, can bypass epithelial barriers and improve local drug concentration, but these traumatic operations are associated with pain, haemorrhage and potential endophthalmitis, making them unsuitable for long-term repeated treatment [8,9,10].

Exosome-like nanovesicles have emerged as promising natural carriers for biological drug delivery [11,12,13,14]. Mammalian stem cell-derived exosomes are widely reported for ocular disease treatment, whereas their clinical translation is restricted by rigorous GMP-level cell culture requirements, high production costs, limited cell passage capacity and unavoidable batch-to-batch differences [15,16,17]. In contrast, plant-derived exosome-like nanovesicles (PELNVs) possess several advantages of stable raw material sources, simple isolation procedures, low cost and good biosafety, without the immunogenic risks of mammalian cell-derived vesicles [18,19,20,21]. Edible plant nanovesicles also contain abundant endogenous bioactive components that provide auxiliary anti-inflammatory and tissue-protective effects, exhibiting great potential for disease intervention [21,22,23,24]. Of various edible PELNVs, Rabexo was chosen herein. Multiple studies have confirmed that Rabexo encapsulates anthocyanins, glucosinolates and antioxidant vitamins, exerting robust antioxidative and anti-inflammatory effects via regulating the Nrf2 and TLR4/NF-κB pathways. Unlike conventional inert plant nanocarriers, Rabexo can concurrently deliver anti-VEGF antibodies and relieve corneal inflammation and oxidative stress, matching the pathological features of alkali-burned cornea [23,25,26].

In this study, Rabexo was prepared and utilized as natural nanocarriers for anti-VEGFA antibody delivery. The antibody-loaded nanovesicles were formulated into standardized topical eye drops using an ophthalmic hydrogel matrix to prolong ocular retention and enhance corneal permeability. This integrated combinatorial delivery system has not been comprehensively documented in prior research. The physicochemical properties, cellular biocompatibility and in vitro anti-angiogenic capacity of the nanoplatform were systematically characterized, and its in vivo therapeutic effects against corneal angiogenesis and subsequent fibrosis were verified in a mouse corneal alkali burn model. This work aims to establish a topical biological delivery strategy for comprehensive treatment of pathological lesions following corneal injury.

## 2. Results

### 2.1. Physicochemical Characterization of Rabexo

**Figure 1:**
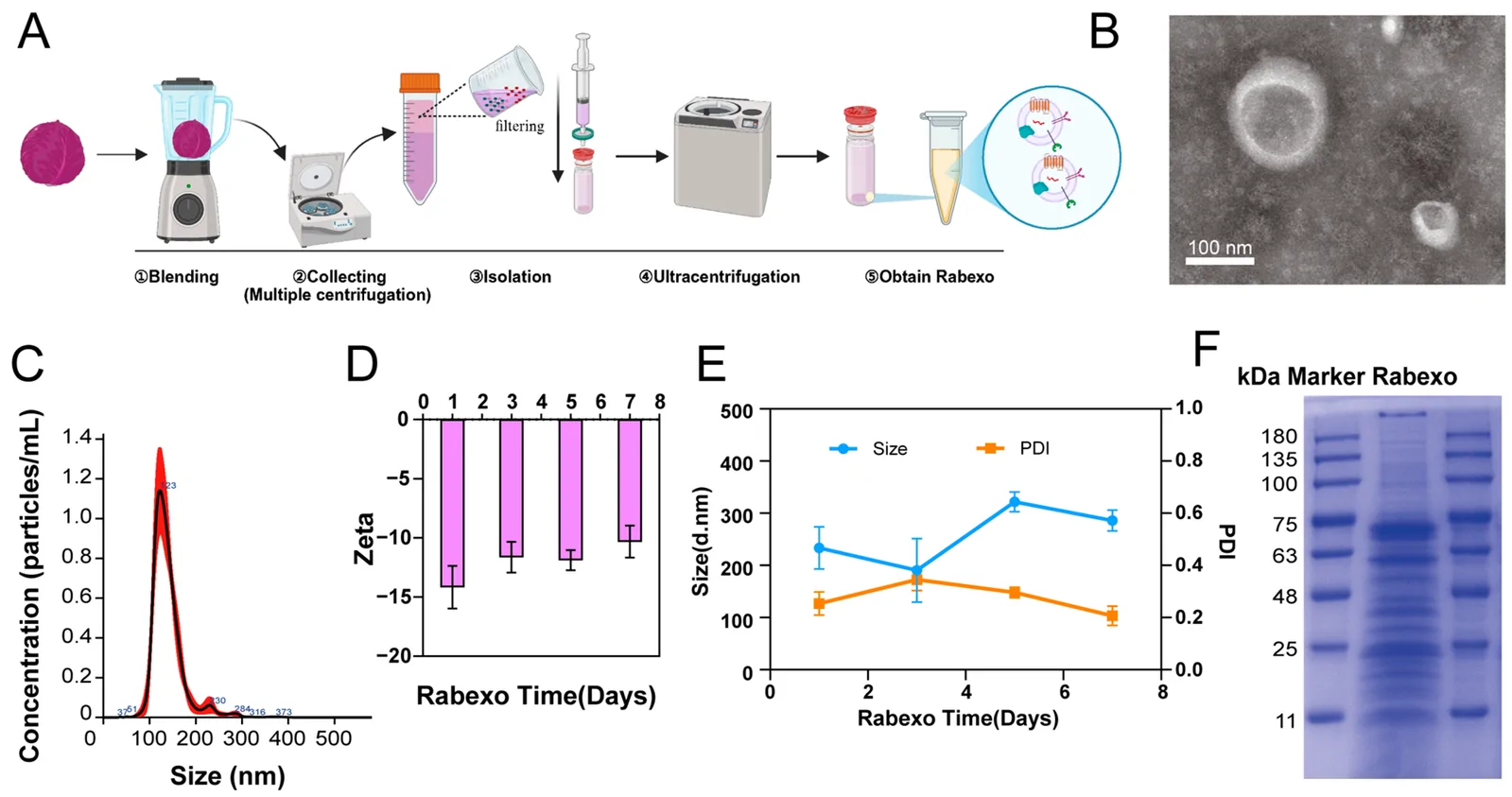
Preparation and characterization of red cabbage-derived exosome-like nanovesicles (Rabexo). (**A**) Schematic diagram of Rabexo isolation process, including blending, centrifugation, and ultrafiltration. (**B**) Rabexo TEM, showing the typical cup-shaped morphology. Scale bar = 100 nm. (**C**) Nanoparticle-tracking analysis (NTA) profile of Rabexo, showing particle concentration as a function of size. (**D**) Zeta potential stability of Rabexo over 7 days of storage. (**E**) Changes in particle size and polydispersity index (PDI) of Rabexo during 7 days of storage. (**F**) SDS-PAGE protein profile of Rabexo. kDa marker is shown on the left.

Red cabbage-derived exosome-like nanovesicles (Rabexo) were successfully isolated via multi-step centrifugation and ultrafiltration (Figure 1A). Transmission electron microscopy (TEM) confirmed that the isolated Rabexo exhibited the typical cup-shaped morphology of exosome-like vesicles, with particle sizes consistent with general exosome characteristics (Figure 1B). Nanoparticle tracking analysis (NTA) revealed a unimodal size distribution, with the main peak at approximately 117.7 ± 2.4 nm (Figure 1C), indicating favorable particle homogeneity. The particle concentration was determined to be (3.80 ± 0.14) × 10^11^ particles/mL, corresponding to an average yield of 3.80 × 10^9^ particles per gram of fresh red cabbage tissue. Dynamic light scattering (DLS) was used to characterize the hydrodynamic properties of Rabexo. The average hydrodynamic diameter was 233.4 ± 40.50 nm, the polydispersity index (PDI) was 0.254 ± 0.04, and the zeta potential was −14.17 ± 1.81 mV. Colloidal stability was assessed by monitoring the zeta potential over 7 consecutive days. Rabexo maintained a stable negative surface charge throughout storage (Figure 1D), suggesting sufficient electrostatic repulsion to prevent particle aggregation. Long-term stability tests further demonstrated that the particle size and PDI of Rabexo remained stable for 7 days (Figure 1E), with no obvious alterations, which supports its application in subsequent biological experiments.

SDS-PAGE analysis was performed to characterize the protein profile of Rabexo (Figure 1F). Distinct protein bands typical of plant-derived nanovesicles were observed, further verifying the successful isolation of Rabexo.

### 2.2. Preparation, Identification, Cellular Uptake and Biosafety of aV-Rabexo

**Figure 2:**
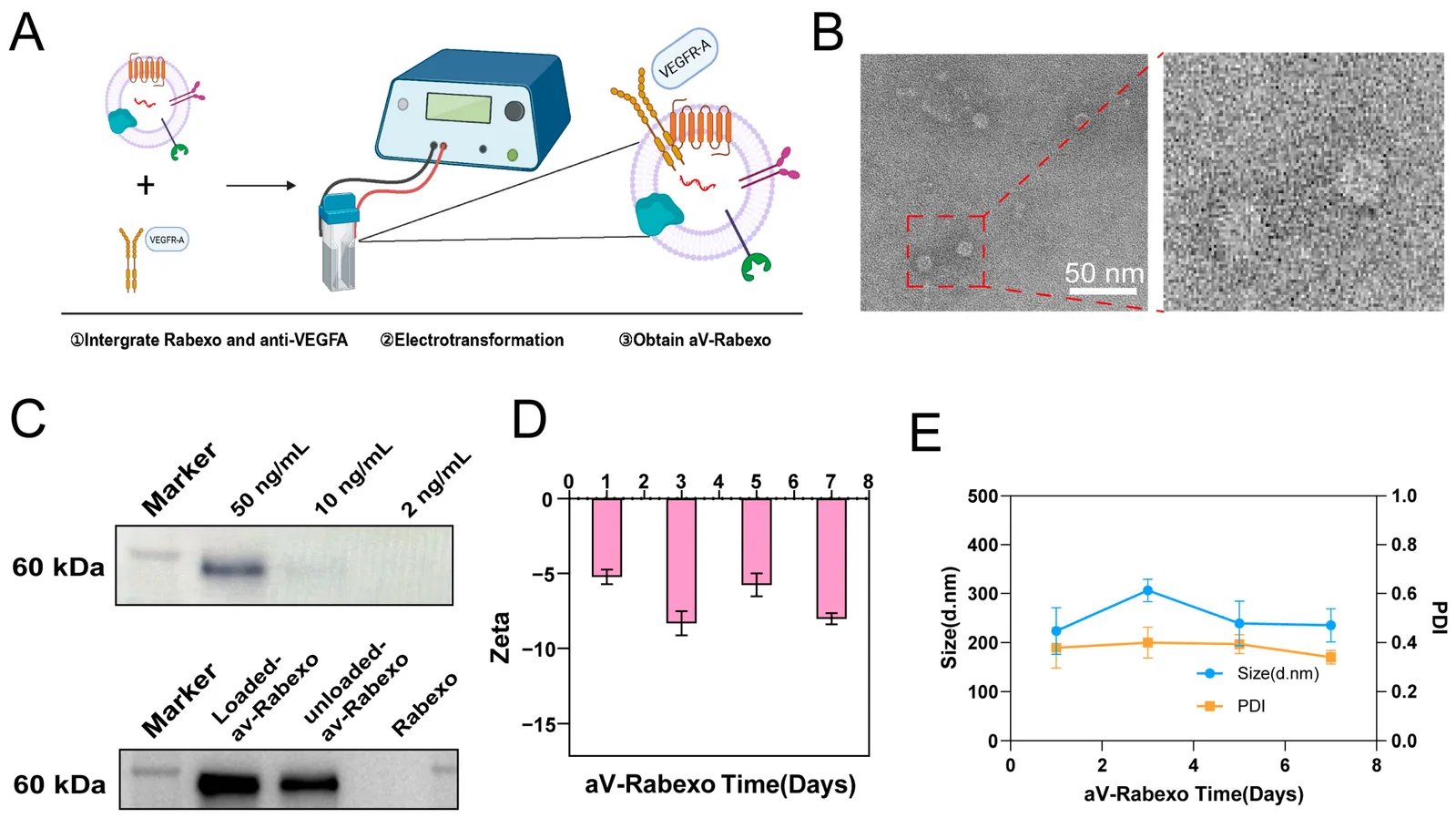
Preparation and characterization of antibody-loaded Rabexo (aV-Rabexo). (**A**) Schematic diagram illustrating the fabrication of aV-Rabexo via electroporation. (**B**) aV-Rabexo TEM, showing typical vesicular morphology. Scale bar = 50 nm. (**C**) WB analysis for anti-VEGFA antibody. Upper panel: standard curve with 50, 10, and 2 ng/mL antibody standards. Lower panel: antibody content in loaded-aV-Rabexo, unloaded-aV-Rabexo, and blank Rabexo. (**D**) Zeta potential stability of aV-Rabexo over 7 days of storage. (**E**) Changes in hydrodynamic size and PDI of aV-Rabexo during 7 days of storage.

Anti-VEGFA antibody-associated Rabexo (aV-Rabexo) was fabricated via electroporation (Figure 2A). TEM results showed that aV-Rabexo retained the intact vesicular morphology of blank Rabexo (Figure 2B), indicating that the electroporation process did not damage vesicle structure.

Western blot (WB) analysis was applied to verify antibody loading. A clear protein band corresponding to anti-VEGFA antibody was detected in the aV-Rabexo group, whereas no band was observed in blank Rabexo, confirming successful antibody encapsulation (Figure 2C). The encapsulation efficiency of anti-VEGFA antibody was calculated to be 40.3%. The colloidal stability of aV-Rabexo was evaluated over 7 days of storage (Figure 2D). The surface potential remained steadily negative, maintaining effective electrostatic repulsion. Long-term stability assessment also showed that the hydrodynamic size and PDI of aV-Rabexo remained stable within 7 days (Figure 2E), with no aggregation or size shift. Collectively, these results demonstrate that aV-Rabexo possesses good monodispersity and colloidal stability, which is suitable for subsequent biological applications.

### 2.3. Cellular Uptake and In Vivo Distribution of aV-Rabexo

**Figure 3:**
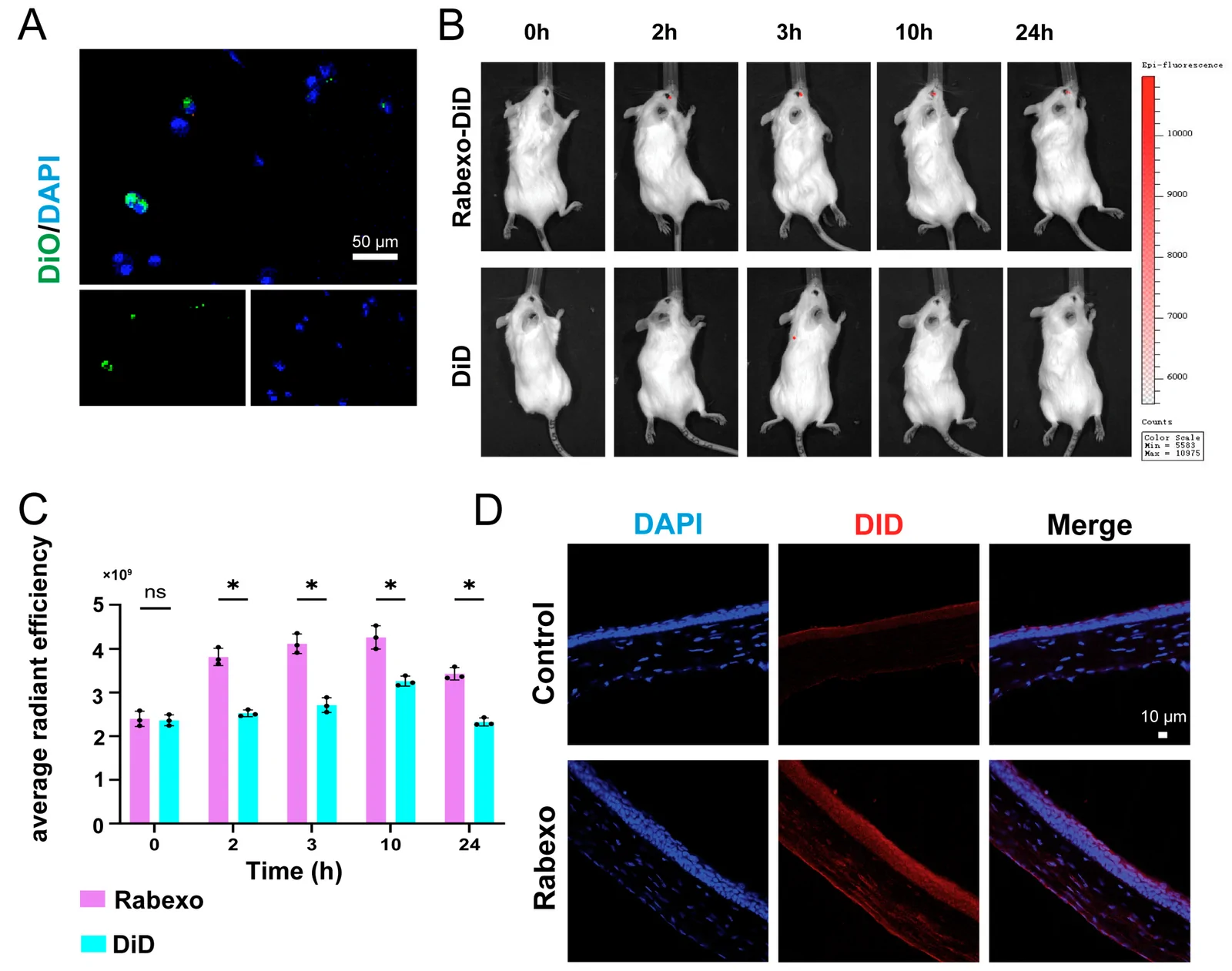
Cellular uptake and in vivo distribution of aV-Rabexo. (**A**) Confocal microscopy images showing the cellular uptake of DiO-labeled Rabexo (green) by cells, with nuclei stained by DAPI (blue). Scale bar = 50 μm. (**B**) In vivo fluorescence imaging of mice after topical administration of DiD-labeled aV-Rabexo and free DiD at 0, 2, 3, 10, and 24 h. (**C**) Quantitative analysis of the average radiant efficiency of aV-Rabexo and DiD control at different time points. Data are presented as mean ± SD (*n* = 3). Statistical analysis was performed using two-way ANOVA followed by Tukey’s multiple comparisons test; *p* = 0.4029 at 0 h, *p* = 0.0038 at 2 h, *p* = 0.0005 at 3 h, *p* = 0.0082 at 10 h, and *p* = 0.0007 at 24 h; (* *p* < 0.05, ns = not significant). (**D**) Fluorescence imaging of corneal frozen sections from mice treated with DiD-labeled aV-Rabexo and control. Nuclei were stained with DAPI (blue), and aV-Rabexo was labeled with DiD (red). Data are presented as mean ± SD (*n* = 3).

We next evaluated cellular uptake and in vivo distribution to verify the ocular targeting capability of aV-Rabexo. Confocal laser scanning microscopy showed that DiO-labeled Rabexo (green) was associated with corneal epithelial cells, with fluorescence signals predominantly distributed in the cytoplasmic region (Figure 3A). For in vivo tracing, DiD-labeled aV-Rabexo was topically administered to mouse eyes. In vivo fluorescence imaging revealed stronger fluorescence signals at ocular sites in the aV-Rabexo group compared with the free DiD group at different time points (Figure 3B). Quantitative analysis of average radiant efficiency showed that the signal of aV-Rabexo peaked at 2 h. Except for the 0 h time point, the radiant efficiency of aV-Rabexo was significantly higher than that of the PBS control group (Figure 3C).

Corneal frozen section fluorescence imaging clearly showed that DiD-labeled aV-Rabexo was distributed throughout the corneal epithelium and stroma, confirming effective tissue penetration and retention of nanovesicles in target tissues (Figure 3D). Intense red fluorescence was detected in the aV-Rabexo-treated group, while only weak background signals were observed in the control group. These results confirm that aV-Rabexo can efficiently accumulate in ocular tissues and penetrate corneal layers, exhibiting favorable targeting and retention properties for local ocular therapy.

### 2.4. Inhibition of Cell Migration and Invasion by aV-Rabexo

**Figure 4:**
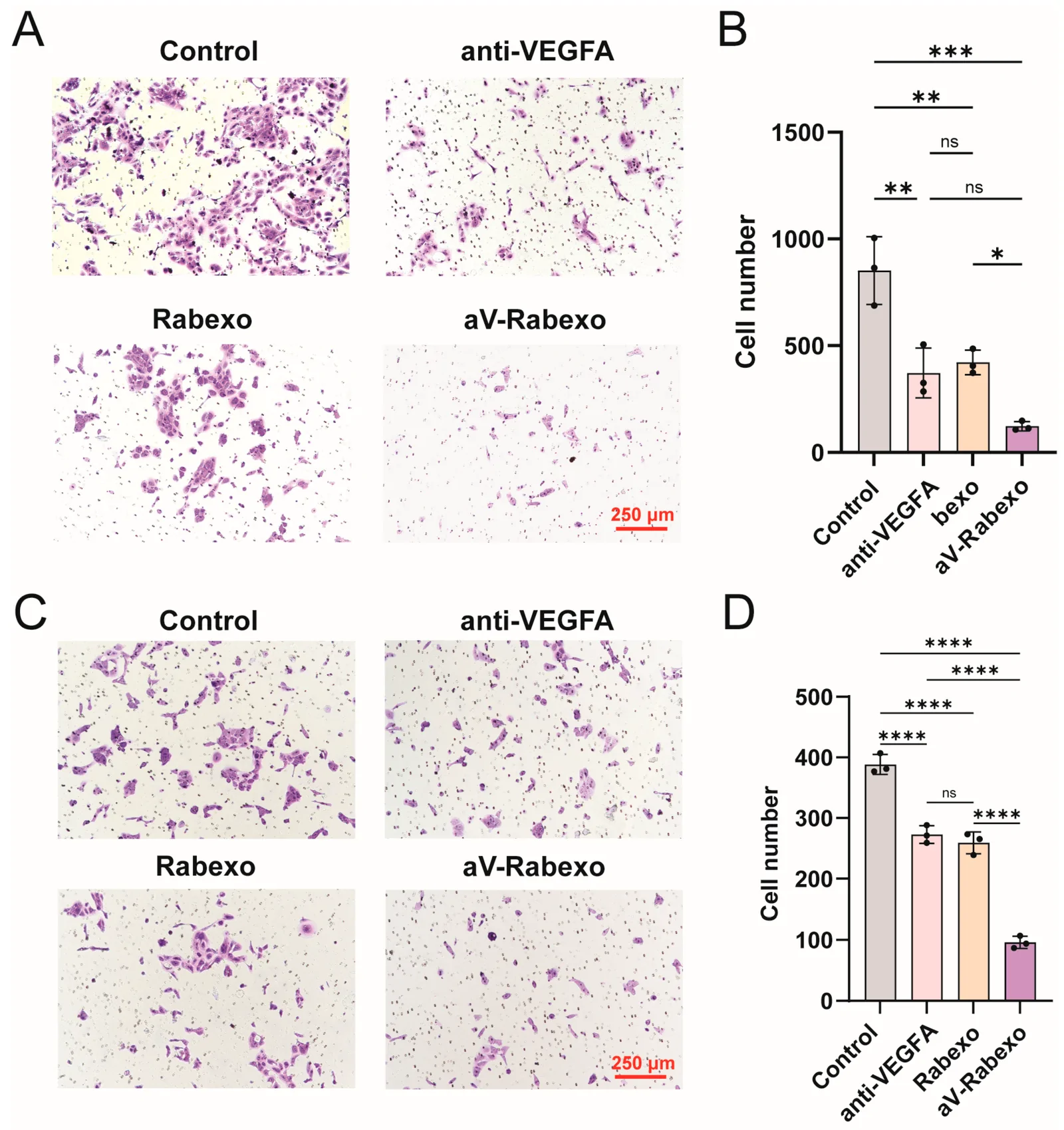
Effects of aV-Rabexo on cell migration and invasion in vitro. (**A**) Representative images of Transwell migration assays for Control, free anti-VEGFA antibody (aV), Rabexo, and aV-Rabexo groups. Scale bar = 250 μm. (**B**) Quantitative analysis of migrated cell numbers in the Transwell assay. Data are presented as mean ± SD (*n* = 3). Control vs. aV, *p* = 0.0020; Control vs. Rabexo, *p* = 0.0040; Control vs. aV-Rabexo, *p* = 0.0001; aV vs. Rabexo, *p* = 0.9335; aV vs. aV-Rabexo, *p* = 0.0702; Rabexo vs. aV-Rabexo, *p* = 0.0309. (**C**) Representative images of cell invasion assays for the four treatment groups. Scale bar = 250 μm. (**D**) Quantitative analysis of invaded cell numbers in the invasion assay. Data are presented as mean ± SD (*n* = 3). Control vs. anti-VEGFA, *p* < 0.0001; Control vs. Rabexo, *p* < 0.0001; Control vs. aV-Rabexo, *p* < 0.0001; anti-VEGFA vs. Rabexo, *p* = 0.6895; anti-VEGFA vs. aV-Rabexo, *p* < 0.0001; Rabexo vs. aV-Rabexo, *p* < 0.0001. Data are presented as mean ± SD (*n* = 3). * *p* < 0.05, ** *p* < 0.01, *** *p* < 0.001, **** *p* < 0.0001; ns = not significant.

Transwell migration and Matrigel-based invasion assays were performed to assess the anti-angiogenic effects of aV-Rabexo. In the migration assay (Figure 4A,B), the number of migrated cells was significantly decreased in all treatment groups relative to the control group: aV (*p* = 0.0020), Rabexo (*p* = 0.0040) and aV-Rabexo (*p* = 0.0001). No significant difference was found between the aV and Rabexo groups (*p* = 0.9335). The aV-Rabexo group exhibited a significantly stronger inhibitory effect than the Rabexo group (*p* = 0.0309), and showed a trend toward enhanced migration inhibition compared with free aV (*p* = 0.0702). Consistent with the migration results, the cell invasion assay (Figure 4C,D) showed that all treatments significantly suppressed cell invasion when compared with the control (all *p* < 0.0001). The anti-VEGFA and Rabexo groups exerted comparable inhibitory effects (*p* = 0.6895), while aV-Rabexo produced significantly stronger inhibition than both free anti-VEGFA and blank Rabexo (both *p* < 0.0001). These in vitro functional experiments demonstrate that aV-Rabexo potently inhibits cell migration and invasion, two critical biological processes during pathological angiogenesis.

### 2.5. Anti-Proliferative and Anti-Angiogenic Effects of aV-Rabexo In Vitro

**Figure 5:**
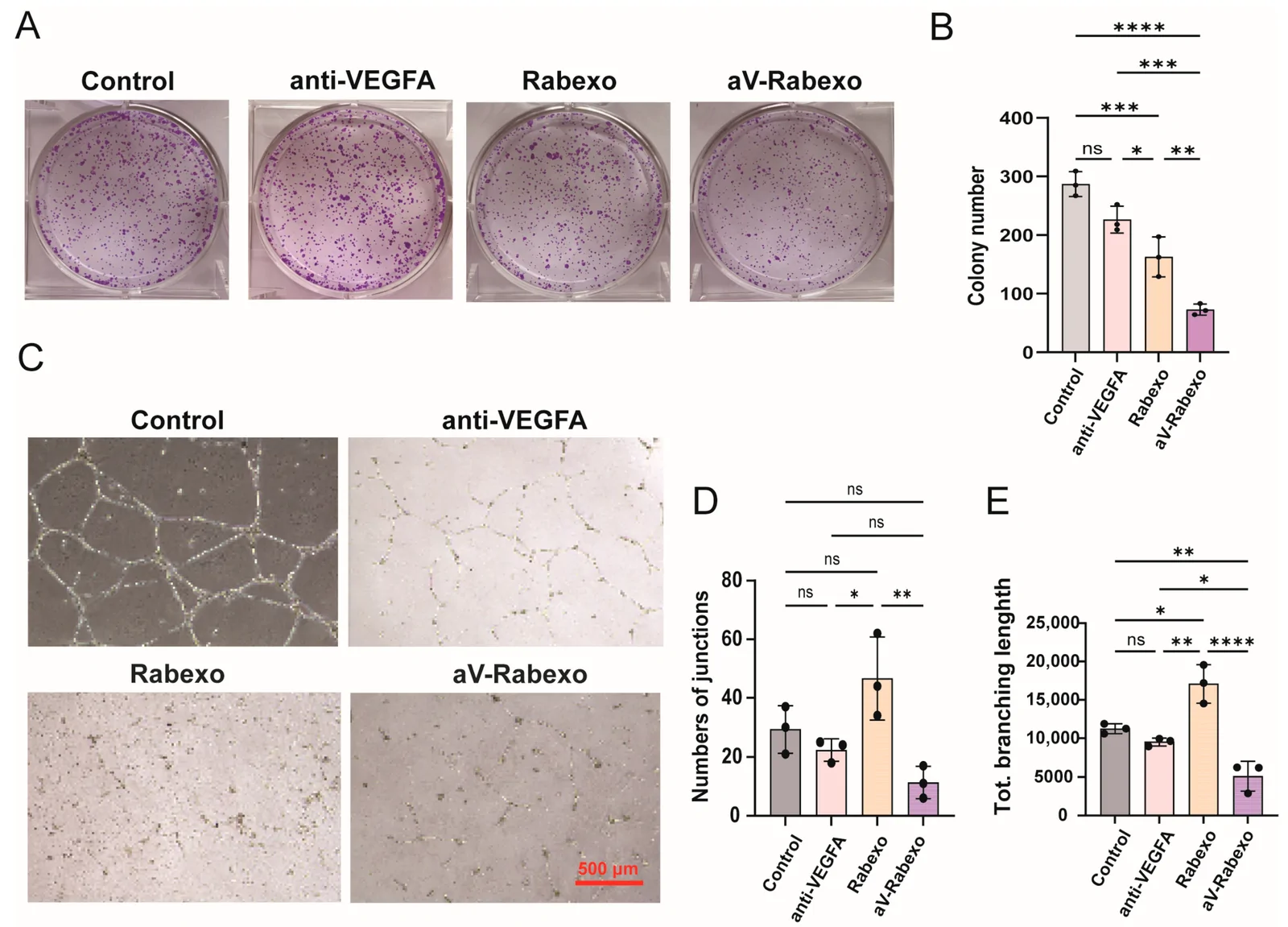
Anti-proliferative and anti-angiogenic effects of aV-Rabexo in vitro. (**A**) Representative images of colony formation assays for Control, free anti-VEGFA antibody, Rabexo, and aV-Rabexo groups. (**B**) Quantitative analysis of colony numbers in the colony formation assay. Data are presented as mean ± SD (*n* = 3). Control vs. anti-VEGFA, *p* = 0.0528; Control vs. Rabexo, *p* = 0.0009; Control vs. aV-Rabexo, *p* < 0.0001; anti-VEGFA vs. Rabexo, *p* = 0.0424; anti-VEGFA vs. aV-Rabexo, *p* = 0.0002; Rabexo vs. aV-Rabexo, *p* = 0.0068. (**C**) Representative images of tube formation assays for the four treatment groups. Scale bar = 500 μm. (**D**) Quantitative analysis of total branching length in the tube formation assay. Data are presented as mean ± SD (*n* = 3). Control vs. anti-VEGFA, *p* = 0.6032; Control vs. Rabexo, *p* = 0.0103; Control vs. aV-Rabexo, *p* = 0.0078; anti-VEGFA vs. Rabexo, *p* = 0.0022; anti-VEGFA vs. aV-Rabexo, *p* = 0.0433; Rabexo vs. aV-Rabexo, *p* < 0.0001. (**E**) Quantitative analysis of the number of junctions in the tube formation assay. Data are presented as mean ± SD (*n* = 3). Control vs. anti-VEGFA, *p* = 0.7679; Control vs. Rabexo, *p* = 0.1522; Control vs. aV-Rabexo, *p* = 0.1339; anti-VEGFA vs. Rabexo, *p* = 0.0388; anti-VEGFA vs. aV-Rabexo, *p* = 0.4649; Rabexo vs. aV-Rabexo, *p* = 0.0051. Data are presented as mean ± SD (*n* = 3). * *p* < 0.05, ** *p* < 0.01, *** *p* < 0.001, **** *p* < 0.0001; ns = not significant.

Colony formation and tube formation assays were further conducted to explore the anti-proliferative and anti-angiogenic activities of aV-Rabexo. The colony formation assay was used to evaluate long-term cell proliferation. The number of colonies gradually decreased across groups, with the lowest count observed in the aV-Rabexo group (Figure 5A,B). Compared with the control group, the free anti-VEGFA group showed a trend toward reduced colony formation (*p* = 0.0528). Both Rabexo and aV-Rabexo significantly inhibited colony formation (*p* = 0.0009 and *p* < 0.0001, respectively). Moreover, aV-Rabexo exhibited significantly greater anti-proliferative capacity than free anti-VEGFA (*p* = 0.0002) and blank Rabexo (*p* = 0.0068). Tube formation assays were performed to evaluate in vitro angiogenic ability. The aV-Rabexo group formed fewer and less complex tubular networks (Figure 5C). Quantitative analysis of total branching length (Figure 5D) revealed that aV-Rabexo significantly reduced tube branching compared with the control (*p* = 0.0078), Rabexo (*p* < 0.0001) and free anti-VEGFA (*p* = 0.0433). Rabexo alone also decreased branching length relative to the control (*p* = 0.0103) and free anti-VEGFA (*p* = 0.0022), but its efficacy was inferior to aV-Rabexo. Quantification of tube junctions (Figure 5E) yielded consistent results. The number of junctions in the aV-Rabexo group was significantly lower than that in the Rabexo group (*p* = 0.0051), while no significant differences were detected when compared with the control and free anti-VEGFA groups (*p* = 0.1339 and *p* = 0.4649, respectively). Taken together, these results indicate that aV-Rabexo exerts stronger inhibitory effects on cell proliferation and in vitro angiogenesis than single treatment with anti-VEGFA or Rabexo.

### 2.6. aV-Rabexo-Gel Suppresses Alkali Burn-Induced Corneal Neovascularization in Mice

**Figure 6:**
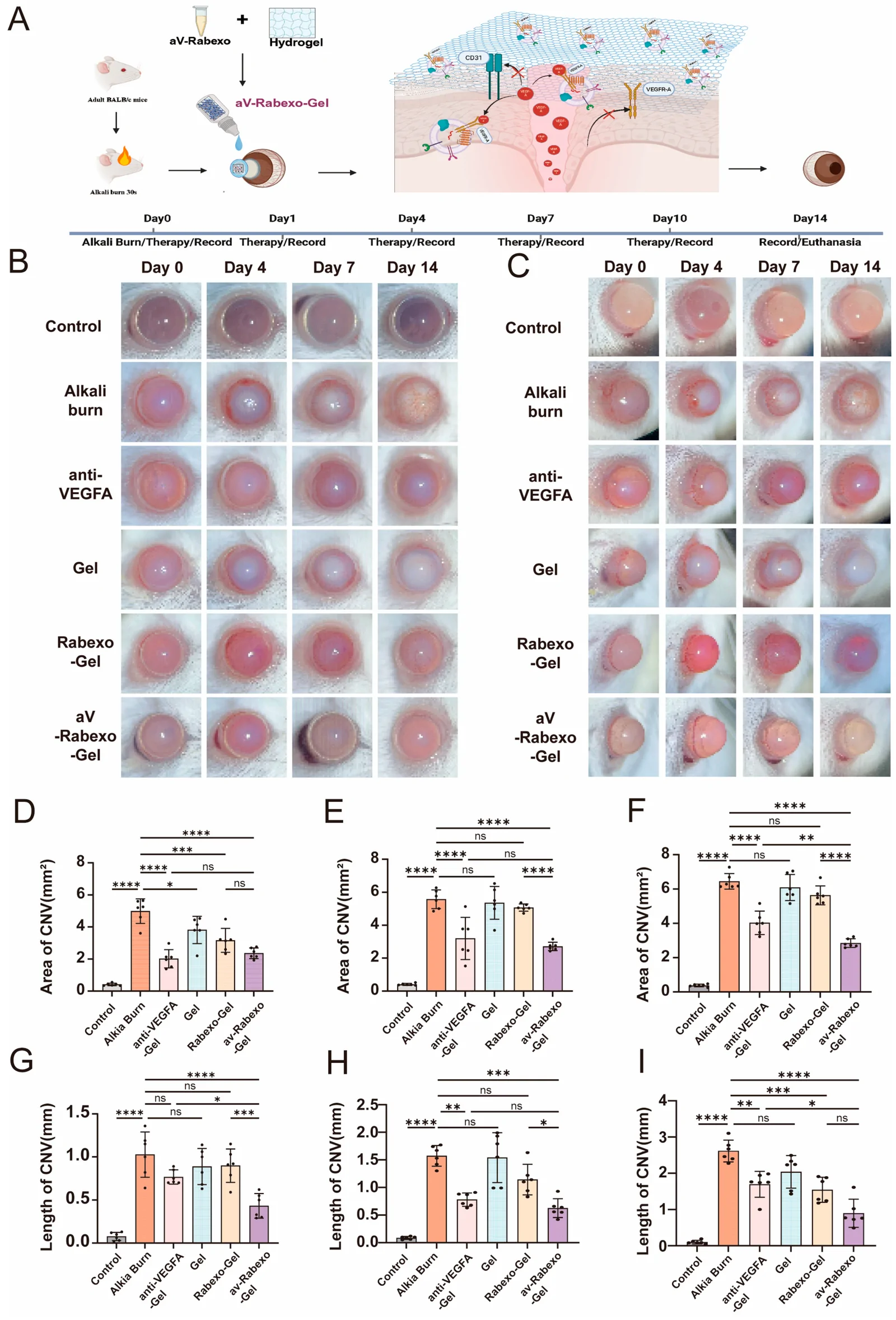
In vivo therapeutic efficacy of aV-Rabexo-loaded hydrogel in an alkali burn-induced corneal neovascularization (CNV) mouse model. (**A**) Schematic illustration of the experimental workflow. (**B**) Representative images of the cornea from a frontal view obtained through slit-lamp examination in the control group, Alkali burn group, anti-VEGFA-Gel group, Gel group, Rabexo-Gel group and aV-Rabexo-Gel group. (**C**) Representative images of the cornea from a lateral view obtained through slit-lamp examination. (**D**–**F**) Quantitative analysis of CNV area (mm^2^) based on B, from left to right: Day 4, Day 7, and Day 14. (**G**–**I**) Quantitative analysis of CNV length (mm) based on C, from left to right: Day 4, Day 7, and Day 14. Data are presented as mean ± SD (*n* = 6). * *p* < 0.05, ** *p* < 0.01, *** *p* < 0.001, **** *p* < 0.0001; ns = not significant.

A mouse model of alkali burn-induced corneal neovascularization (CNV) was established to evaluate the in vivo therapeutic efficacy of aV-Rabexo-loaded hydrogel (aV-Rabexo-Gel). Mice were divided into six groups: normal control, alkali burn model, blank hydrogel (Gel), Rabexo-loaded hydrogel (Rabexo-Gel), anti-VEGFA-loaded hydrogel (anti-VEGFA-Gel) and aV-Rabexo-Gel. Slit-lamp microscopy was used to dynamically observe CNV growth (Figure 6B,C). The area and length of pathological neovessels were quantified on Days 4, 7 and 14 after modeling.

On Day 4 after alkali burn, obvious vascular sprouting was observed at the corneal limbus in the alkali burn group, with neovessels extending toward the central cornea. The progression of CNV in the Gel and Rabexo-Gel groups was slightly slower than that in the model group, and no significant difference was found in the Gel group. Both aV-Rabexo-Gel and anti-VEGFA-Gel significantly inhibited CNV formation, with significantly reduced neovascular area compared with the alkali burn group (*p* < 0.0001, Figure 6D). The neovascular length in the aV-Rabexo-Gel group was remarkably shorter than that in the alkali burn group (*p* < 0.0001) and the anti-VEGFA-Gel group (*p* = 0.0244, Figure 6G).

On Day 7, intergroup differences became more evident. Quantitative results of CNV area and length revealed a clear therapeutic gradient: aV-Rabexo-Gel > anti-VEGFA-Gel > Rabexo-Gel > Gel > alkali burn model (Figure 6E,H). The aV-Rabexo-Gel group showed smaller CNV area and length than the anti-VEGFA-Gel group (without statistical significance), and presented significant differences compared with all other groups.

On Day 14, the above therapeutic gradient remained stable. Neovessels nearly covered the entire cornea in the alkali burn group. The blank Gel showed limited therapeutic effects, with no significant difference from the model group at all time points. Although antiVEGFA-Gel and Rabexo-Gel partially suppressed CNV, their efficacy was significantly weaker than aV-Rabexo-Gel. On day 14 after modeling, aV-Rabexo-Gel significantly inhibited alkali burn-induced corneal neovascularization, reducing CNV area by 56.0% and length by 64.3% compared with the alkali burn group, and reducing CNV area by 29.5% and length by 45.2% compared with the anti-Vegfa-Gel group. Partial regression of neovessels was even observed in some corneas of the aV-Rabexo-Gel group (Figure 6B,C). In summary, the hydrogel formulation loaded with aV-Rabexo showed significantly enhanced therapeutic effects against alkali burn-induced pathological CNV in vivo.

### 2.7. aV-Rabexo-Gel Alleviates Corneal Fibrosis and Regulates Related Protein Expression

Histopathological and molecular biological analyses of corneal tissues were performed to explore the underlying therapeutic mechanisms of aV-Rabexo-Gel.

**Figure 7:**
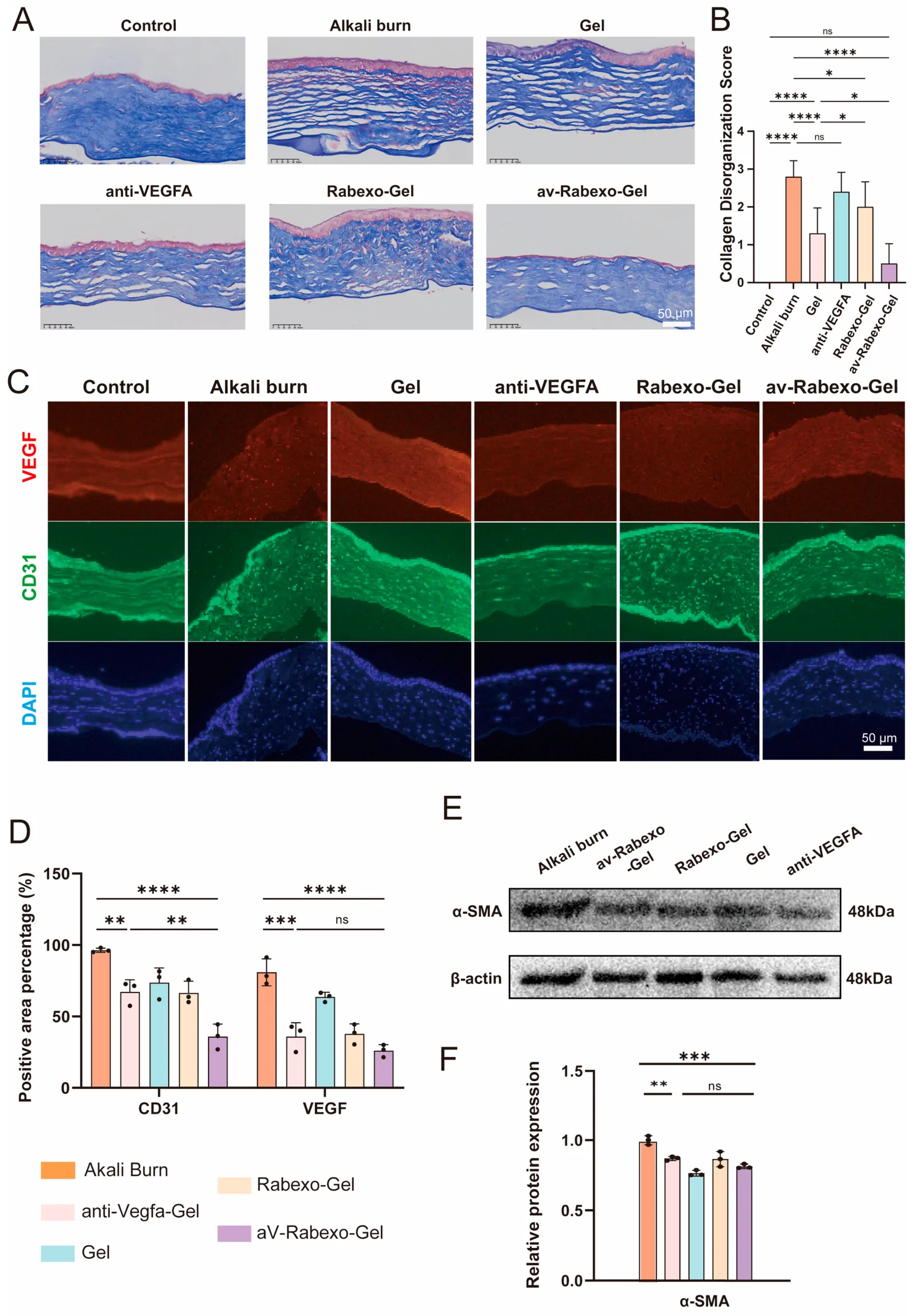
Histopathological and molecular evaluation of corneal tissue after treatment with aV-Rabexo-loaded hydrogel. (**A**) Representative Masson’s trichrome staining of corneal sections showing collagen organization in different groups. Scale bars are included in the original images. (**B**) Quantitative analysis of collagen disorganization scores based on Masson’s trichrome staining. Data are presented as mean ± SD (*n* = 3). (**C**) Representative immunofluorescence images of corneal sections stained for VEGF (red), CD31 (green), and DAPI (blue). (**D**) Quantitative analysis of CD31 and VEGF positive area percentages from immunofluorescence staining. Data are presented as mean ± SD (*n* = 3). (**E**) Western blot analysis of α-SMA protein expression in corneal tissues. β-actin was used as the loading control. (**F**) Quantitative analysis of relative protein expression levels from Western blot. Data are presented as mean ± SD (*n* = 3). * *p* < 0.05, ** *p* < 0.01, *** *p* < 0.001, **** *p* < 0.0001; ns = not significant.

Masson’s trichrome staining showed severe disorder of collagen fibers in the alkali burn group, while collagen arrangement was obviously improved in the aV-Rabexo-Gel group (Figure 7A). Collagen disorganization was semi-quantitatively scored by two independent blinded observers according to a standardized scale. Quantitative results confirmed that collagen damage was significantly alleviated in the aV-Rabexo-Gel group relative to the alkali burn control (Figure 7B).

Immunofluorescence staining was used to detect the expression of CD31 (endothelial cell marker) and VEGF (key angiogenic factor). Strong positive signals were observed in the alkali burn group, which were obviously attenuated after aV-Rabexo-Gel treatment (Figure 7C). Quantitative analysis (Figure 7D) showed that the CD31-positive area in the aV-Rabexo-Gel group was significantly lower than that in the control (*p* < 0.0001) and anti-VEGFA groups (*p* = 0.0055). For VEGF expression, aV-Rabexo-Gel induced a significant reduction compared with the control (*p* < 0.0001), and no significant difference was observed between the aV-Rabexo-Gel and anti-VEGFA groups (*p* = 0.4823).

Western blot analysis further verified the above results (Figure 7E,F). CD31 expression was significantly downregulated in the anti-VEGFA group versus the control (*p* = 0.0067), and was further decreased in the aV-Rabexo-Gel group (*p* = 0.0011 vs. anti-VEGFA). The expression of α-SMA was significantly inhibited in both aV-Rabexo-Gel (*p* = 0.0002) and anti-VEGFA groups (*p* = 0.0029) compared with the control, with no significant difference between the two treatment groups (*p* = 0.2527). Notably, VEGF expression was significantly suppressed in the aV-Rabexo-Gel group when compared with the control (*p* < 0.0001) and anti-VEGFA groups (*p* < 0.0001). The free anti-VEGFA group only showed a trend toward decreased VEGF expression (*p* = 0.0795 vs. control).

Collectively, these results demonstrate that aV-Rabexo-Gel ameliorates alkali burn-induced corneal injury by inhibiting angiogenesis, downregulating VEGF expression and protecting collagen structure, with significantly greater overall efficacy than free anti-VEGFA monotherapy.

## 3. Discussion

Topical delivery of macromolecular biological agents remains a major challenge in ophthalmic therapy due to the combined barriers of rapid tear clearance and the tight corneal epithelial barrier. Invasive intraocular injections can improve local drug concentration but carry risks of trauma, infection and poor patient compliance for long-term use [2,27]. In the present study, we constructed a topical ocular delivery system using red cabbage-derived exosome-like nanovesicles (Rabexo) to deliver anti-VEGFA antibody. This platform achieves simultaneous intervention against corneal angiogenesis and secondary stromal fibrosis, two core pathological changes after severe corneal injury. Different from most studies that only focus on corneal neovascularization, this work pays attention to the overall pathological progression of injured cornea, which is more consistent with real clinical scenarios.

Corneal frozen section fluorescence results directly confirmed that Rabexo could penetrate the corneal epithelial barrier and distribute within corneal stroma. The uptake of plant-derived exosome-like nanovesicles into target cells may involve three distinct mechanisms: fusion with the target cell membrane, internalization through endocytosis, and binding to receptors on the target cell membrane, as reviewed by Zhao et al. [28]. Combined with the PVA/HA ophthalmic hydrogel, the nanovesicles achieved prolonged retention on the ocular surface and sustained drug release effects, effectively overcoming the key pharmacokinetic obstacles for topical macromolecular administration. In vitro experiments using human corneal epithelial cells and human umbilical vein endothelial cells (HUVECs, purchased from ATCC) verified that Rabexo significantly enhanced the anti-migration, anti-invasion, anti-proliferation and anti-tube formation abilities of the anti-VEGFA antibody. In the mouse alkali burn corneal injury model, aV-Rabexo-Gel substantially reduced the area and length of pathological corneal neovascularization, restored the disordered collagen structure, and downregulated the expression of angiogenic markers (VEGFA, CD31) and the fibrotic marker (α-SMA), with significantly greater in vivo therapeutic efficacy than the free antibody hydrogel.

Compared with mammalian stem cell-derived exosomes, Rabexo possesses superior clinical translational potential. Mammalian exosomes require rigorous GMP cell culture, accompanied by high production costs, obvious batch fluctuations and inherent immunogenic risks, which greatly restrict their large-scale clinical application [29,30]. In contrast, Rabexo is isolated from widely cultivated red cabbage through simple centrifugation and ultrafiltration procedures. It boasts low cost, favorable batch consistency and excellent ocular biocompatibility without immune adverse reactions [26,31,32]. Rabexo contains endogenous bioactive components that may contribute to the overall therapeutic outcome. It is plausible that these intrinsic activities could complement the anti-angiogenic effect of the loaded antibody; however, the precise nature of this interaction requires further investigation. When compared with exosome-like nanovesicles derived from ginger, citrus and regular cabbage, Rabexo accumulates abundant anthocyanins and thus presents stronger anti-inflammatory and antioxidant activities. Instead of merely acting as an inert drug carrier, Rabexo alleviates corneal inflammation and oxidative stress while transporting antibodies, perfectly matching the complex pathological conditions of alkali-burned cornea. Its moderate particle size also enables efficient corneal epithelial penetration for ideal ocular surface targeted delivery.

The PVA/HA hydrogel adopted in this study is a mature, clinically compatible ophthalmic carrier with good biocompatibility and tear film compatibility [33]. Notably, this hydrogel matrix is a commercially available material that can be easily purchased from regular suppliers, featuring simple preparation procedures and good manufacturability for large-scale industrial production. It can form a protective film on the ocular surface to resist tear dilution and loss. In this study, pure PVA/HA hydrogel showed no obvious independent anti-angiogenic or anti-fibrotic effects, and only acted as a sustained-release and retention auxiliary matrix. The significant efficacy gap between aV-Rabexo-Gel and free antibody hydrogel fully proves that the core therapeutic advantages originate from the unique penetration capability, bioactivity protection and potential synergistic effect of Rabexo nanocarriers, rather than the hydrogel itself. As a topical preparation, hydrogel eye drops are more suitable for long-term repeated administration and have higher patient acceptance than microneedles and implantable sustained-release devices [34,35].

Mechanistically, excessive VEGFA expression after corneal alkali burn initiates pathological angiogenesis, activates inflammatory cascades and promotes fibroblast-myofibroblast transition, eventually leading to irreversible corneal fibrosis and scarring [36,37,38]. Traditional topical anti-VEGFA formulations fail to efficiently cross the corneal barrier and are rapidly cleared by tears, resulting in discontinuous VEGFA blockade and inability to curb the progression of inflammation and fibrosis [8]. Benefiting from the advantages of nanocarriers, the aV-Rabexo system achieves fluorescence-associated distribution and long-term local retention, realizing continuous and stable inhibition on VEGFA signaling. Meanwhile, the endogenous active substances of Rabexo can alleviate inflammatory infiltration and oxidative stress damage, which may help regulate the progression of angiogenesis, inflammation and fibrosis. The simultaneous reduction in both angiogenic (VEGFA, CD31) and fibrotic (α-SMA) biomarkers suggests that aV-Rabexo-Gel may offer broader therapeutic benefits compared with strategies targeting angiogenesis alone. We propose that aV-Rabexo-Gel may indirectly improve the corneal microenvironment by suppressing VEGFA-driven angiogenesis and fibrotic progression; however, whether the formulation possesses direct anti-inflammatory effects requires further experimental validation.

In conclusion, this study successfully developed a biocompatible, spatiotemporally controlled delivery system, aV-Rabexo-Gel, based on red cabbage-derived exosome-like nanovesicles. Relying on the synergistic strategy of nanovesicle-mediated delivery, antibody blockade and hydrogel-mediated sustained retention, this platform effectively reverses alkali burn-induced corneal angiogenesis and fibrosis by regulating the expression of VEGFA, CD31 and α-SMA. With stable raw material sources, low cost, non-immunogenicity and scalable production, this plant-derived nanocarrier system has competitive prospects for clinical application in the treatment of ocular surface pathological lesions after injury.

## 4. Materials and Methods

### 4.1. Isolation and Purification of Red Cabbage-Derived Exosome-like Nanovesicles (Rabexo)

Fresh red cabbage (*Brassica oleracea* var. capitata *F. rubra*) was obtained from farms in Yunnan, China. The red cabbage was washed three times with distilled water, chopped into small chunks, and placed in a juicer (Joyoung Z8-V82, Joyoung Co., Ltd., Jinan, China). An appropriate amount of PBS was added, and juicing was performed at 50 rpm for approximately 10 min. The juice was then subjected to differential centrifugation: 500× *g* for 30 min, 2000× *g* for 30 min, and 10,000× *g* for 60 min at 4 °C (5424R Refrigerated Centrifuge, Eppendorf AG, Hamburg, Germany) to remove cell debris and large organelles. The resulting supernatant was ultracentrifuged at 120,000× *g* for 90 min at 4 °C (Optima MAX-XP Ultracentrifuge, Beckman Coulter Inc., Brea, CA, USA). After discarding the supernatant, the pellet was resuspended in sterile PBS and subjected to an additional ultracentrifugation wash at 120,000× *g* for 90 min. The final pellet was resuspended in sterile PBS, aliquoted, and stored at −80 °C for subsequent experiments. The protein concentration of the isolated Rabexo was determined using a BCA assay kit (P0010, Beyotime Biotechnology, Shanghai, China) according to the manufacturer’s instructions.

### 4.2. Physicochemical Characterization of Rabexo

The morphology of Rabexo was observed by transmission electron microscopy (TEM, HT7800, Hitachi, Ltd., Tokyo, Japan). Briefly, 10 μL of Rabexo suspension (diluted to approximately 1 × 10^9^ particles/mL with PBS) was placed on a formvar-coated copper grid and allowed to adsorb for 1–2 min. Excess liquid was removed with filter paper (Whatman, Maidstone, Kent, UK), and the grid was negatively stained with 10 μL of phosphotungstic acid solution (1%, pH 7.0) for 60 s. After air-drying at room temperature, samples were observed under TEM at an accelerating voltage of 80–120 kV. Particle size distribution, concentration, and polydispersity index (PDI) were measured via nanoparticle tracking analysis (NTA, NanoSight NS300, Malvern Panalytical Ltd., Malvern, UK). For NTA measurement, samples were diluted with PBS to 1 × 10^8^ particles/mL and measured in triplicate with a camera level of 15 and a detection threshold of 5. Dynamic light scattering (DLS, Zetasizer Nano ZEN3700, Malvern Panalytical Ltd., Malvern, UK) was used to detect zeta potential in PBS (pH 7.4) at 25 °C with three replicate measurements per sample. The colloidal stability of Rabexo was monitored for 7 consecutive days by detecting changes in particle size, PDI and zeta potential at 0, 1, 3, 5, and 7 days. SDS-PAGE (12% polyacrylamide gel) was performed to analyze the endogenous protein components of Rabexo. Briefly, 30 μg of protein was mixed with 5× loading buffer, boiled for 10 min, separated at 120 V for 90 min, and stained with Coomassie Brilliant Blue R-250 (Beyotime Biotechnology, Shanghai, China).

### 4.3. Preparation and Characterization of aV-Rabexo

Anti-VEGFA antibody (A00045-2, Boster Biological Technology, Ltd., Pleasanton, CA, USA) was loaded into Rabexo via electroporation. An electroporation buffer containing 100 mM trehalose (T9531, Sigma-Aldrich, Inc., St. Louis, MO, USA) in PBS (pH 7.4) was prepared. Rabexo (100 μg protein) and the antibody (20 μg) were mixed, and the volume was adjusted to 800 μL with the electroporation buffer. The mixture was transferred to a 4 mm gap cuvette (1652088, Bio-Rad Laboratories, Inc., Hercules, CA, USA) and electroporated using a Gene Pulser Xcell system (Bio-Rad Laboratories, Inc., Hercules, CA, USA) under optimized conditions: 200 V, 10 ms pulse length, 5 pulses, and a 1 s pulse interval. After electroporation, the sample was placed on ice for 30 min to allow membrane recovery. To remove unencapsulated free antibody, the electroporated mixture was diluted with PBS to 4 mL and transferred to a 100 kDa ultrafiltration tube (UFC8100, MilliporeSigma, Burlington, MA, USA). The tube was centrifuged at 2500× *g* for 15 min at 4 °C. The retentate (aV-Rabexo) was washed twice with PBS by repeating the ultrafiltration step, and the flow-through fractions containing free antibody were collected. The purified aV-Rabexo was resuspended in PBS and stored at −80 °C for further use. The morphological integrity of aV-Rabexo was observed by TEM using the same protocol as described in Section 4.2. The colloidal stability of aV-Rabexo was evaluated by monitoring hydrodynamic size, PDI, and zeta potential over 7 consecutive days. Antibody loading was verified by Western blot as described in Section 4.14. Encapsulation efficiency was calculated based on a semi-quantitative Western blot analysis, where a standard curve was generated using serial dilutions of VEGF standard (50, 10, and 2 ng/mL). The grayscale values of the loaded-aV-Rabexo and unloaded-aV-Rabexo bands were interpolated into the standard curve to determine antibody concentrations, and the encapsulation efficiency was calculated as the percentage of antibody retained in the loaded fraction relative to the total recovered antibody. The morphological integrity of aV-Rabexo was observed by TEM. The colloidal stability of aV-Rabexo was evaluated by monitoring hydrodynamic size, PDI, and zeta potential over 7 consecutive days to confirm its stability for subsequent biological experiments.

### 4.4. Preparation of aV-Rabexo-Gel Topical Formulation

The aV-Rabexo suspension was adjusted to a final working concentration of 2 × 10^9^ particles/mL with sterile PBS (Sinopharm Chemical Reagent Co., Ltd., Shanghai, China) and mixed uniformly with pre-warmed (37 °C, 10 min) PVA/HA composite hydrogel (catalog No. R-SNJ-0285; Xi’an Ruixi Biotechnology Co., Ltd., Xi’an, China). The final formulation contained approximately 1 μg of anti-VEGFA antibody per 20 μL of the prepared gel. The mixture was incubated at 37 °C for an additional 10 min to allow gelation, forming a uniform transparent ophthalmic gel. Blank hydrogel (without exosomes or antibody) and blank Rabexo-Gel (containing unloaded exosomes) were prepared using the same procedure as controls. All formulations were freshly prepared on the day of administration.

### 4.5. Cell Culture

Human corneal epithelial cells (HCECs) and human umbilical vein endothelial cells (HUVECs) were purchased from the American Type Culture Collection (ATCC, Manassas, VA, USA). HCECs were cultured in DMEM/F-12 (11320033, Gibco, Thermo Fisher Scientific, Inc., Waltham, MA, USA) supplemented with 10% fetal bovine serum (FBS, 10099-141, Gibco, Thermo Fisher Scientific, Inc., Waltham, MA, USA) and 1% penicillin-streptomycin solution (15140122, Gibco, Thermo Fisher Scientific, Inc., Waltham, MA, USA). HUVECs were maintained in endothelial cell medium (ECM, 1001, ScienCell Research Laboratories, Inc., Carlsbad, CA, USA) containing 10% FBS, 1% endothelial cell growth supplement (1052, ScienCell Research Laboratories, Inc., Carlsbad, CA, USA) and 1% penicillin-streptomycin solution. Cells were passaged at 80–90% confluence using 0.25% trypsin-EDTA (25200056, Gibco, Thermo Fisher Scientific, Inc., Waltham, MA, USA). All cells were incubated at 37 °C in a humidified atmosphere with 5% CO_2_. Cells at passages 3–8 were used for all experiments. Culture medium was replaced every 2–3 days.

### 4.6. Cellular Uptake Assay

The association of Rabexo with HCECs was examined using confocal microscopy. Rabexo was first labeled with the green fluorescent dye DiO (C1419S, Beyotime Biotechnology, Shanghai, China) according to the manufacturer’s instructions (5 μM, 37 °C, 30 min), followed by ultrafiltration (100 kDa) to remove unbound dye. HCECs were seeded into confocal dishes at a density of 1 × 10^5^ cells per dish and cultured overnight to allow attachment. The cells were then incubated with DiO-labeled Rabexo (100 μL, 2 × 10^9^ particles/mL) at 37 °C for 4 h. After incubation, cells were washed three times with PBS to remove unbound exosomes, and then fixed with 4% paraformaldehyde (P0099, Beyotime Biotechnology, Shanghai, China) for 15 min at room temperature. Nuclei were counterstained with DAPI (62248, 1 μg/mL, Thermo Fisher Scientific, Inc., Waltham, MA, USA) for 10 min in the dark. Fluorescent images were captured using a confocal laser scanning microscope (LSM 880, Carl Zeiss AG, Oberkochen, Germany) with the following settings: DiO excitation at 488 nm/emission at 500–550 nm; DAPI excitation at 405 nm/emission at 420–480 nm, 40× objective, and identical acquisition parameters for all groups.

### 4.7. In Vivo Distribution Study

For in vivo tracing, DiD-labeled Rabexo (prepared by incubating Rabexo with 5 μM DiD (D7757, Thermo Fisher Scientific, Inc., Waltham, MA, USA) at 37 °C for 30 min, followed by ultrafiltration to remove free dye) was topically administered (20 μL) to mouse ocular surfaces. Free DiD solution was used as the control at the same concentration. In vivo fluorescence imaging was performed using an IVIS^®^ Spectrum imaging system (PerkinElmer, Inc., Waltham, MA, USA) at 0, 2, 3, 10, and 24 h post-administration to observe the real-time distribution and retention of Rabexo. The imaging parameters were set as follows: excitation wavelength at 644 nm, emission wavelength at 663 nm, exposure time: auto, field of view: D, binning: 4, and f/stop: 2. Mice were anesthetized using 2% isoflurane (RWD Life Science Co., Ltd., Shenzhen, China) in oxygen (flow rate 0.8 L/min) during imaging. The average radiant efficiency (p/s/cm^2^/sr) in the ocular region was quantitatively analyzed using Living Image software (version 4.4, PerkinElmer, Inc., Waltham, MA, USA). After in vivo fluorescence tracing, mouse eyeballs were enucleated and embedded in optimal cutting temperature (OCT) compound (Tissue-Tek^®^ O.C.T. Compound, catalog 4583, Sakura Finetek Japan Co., Ltd., Tokyo, Japan) for frozen section preparation. Continuous corneal frozen sections with a thickness of 10 μm were sliced at −20 °C using a cryostat (CM1950, Leica Biosystems Nussloch GmbH, Nussloch, Germany). Sections were fixed with 4% paraformaldehyde, permeabilized with 0.2% Triton X-100, blocked with 5% BSA, followed by nuclear staining with DAPI (1 μg/mL, 5 min). The distribution of DiD-labeled Rabexo in each layer of the cornea was observed and photographed using a confocal laser scanning microscope (LSM 880, Carl Zeiss AG, Oberkochen, Germany) with the following settings: DiD excitation at 644 nm/emission at 663 nm; DAPI excitation at 405 nm/emission at 420–480 nm. This procedure was used to verify the penetration and spatial distribution of nanovesicles in corneal tissues.

### 4.8. Cell Migration and Invasion Assays

HCECs at passage 4–6 were used for migration and invasion assays. Transwell chambers (3422, 8.0 μm pore size, Corning, Inc., Corning, NY, USA) were used. For the migration assay, HCECs were serum-starved for 12 h prior to the experiment. Cells were seeded in the upper chamber at a density of 5 × 10^4^ cells per well in 200 μL of serum-free medium, while 600 μL of complete medium (with 10% FBS) was added to the lower chamber as a chemoattractant. All treatments were used at matched concentrations. The following treatment groups were tested: control (PBS), free anti-VEGFA (10 μg/mL, ab46154, Abcam plc, Cambridge, UK), Rabexo (2 × 10^9^ particles/mL, matching the exosome concentration in the aV-Rabexo group), and aV-Rabexo (antibody concentration 10 μg/mL; exosome concentration 2 × 10^9^ particles/mL). After 24 h of incubation, non-migrated cells on the upper side of the membrane were removed with a cotton swab. Migrated cells on the lower side were fixed with 4% paraformaldehyde for 15 min, stained with 0.1% crystal violet solution (C0121, Beyotime Biotechnology, Shanghai, China) for 20 min, rinsed with distilled water, and counted under an inverted microscope (DMi8, Leica Microsystems GmbH, Wetzlar, Germany) at 200× magnification. Five random fields per well were counted, and the average number of migrated cells was calculated. For the invasion assay, Transwell inserts were pre-coated with 50 μL of growth factor-reduced Matrigel (356234, 1:8 dilution in serum-free medium, Corning, Inc., Corning, NY, USA) and allowed to solidify at 37 °C for 30 min. Cells (1 × 10^5^ cells/well in 200 μL serum-free medium) were seeded in the upper chamber, and 600 μL of complete medium was added to the lower chamber. After 48 h of incubation, invasive cells were fixed, stained, and quantified using the same protocol as the migration assay. All experiments were performed in triplicate.

### 4.9. Colony Formation Assay

HCECs at passage 4–6 were seeded into 6-well plates at a density of 500 cells per well and cultured overnight. All treatments were used at matched concentrations. The following treatment groups were tested: control (PBS), free anti-VEGFA (10 μg/mL, ab46154, Abcam plc, Cambridge, UK), Rabexo (2 × 10^9^ particles/mL, matching the exosome concentration in the aV-Rabexo group), and aV-Rabexo (antibody concentration 10 μg/mL; exosome concentration 2 × 10^9^ particles/mL). After 14 days of culture with medium and treatment replenishment every 3 days, cell colonies were washed with PBS, fixed with 4% paraformaldehyde for 30 min, and stained with 0.1% crystal violet for 20 min. Colonies containing ≥ 50 cells were counted under a microscope. The colony formation efficiency was calculated as: (number of colonies/number of seeded cells) × 100%. All experiments were performed in triplicate.

### 4.10. Tube Formation Assay

HUVECs at passage 4–6 were used for the tube formation assay. Growth factor-reduced Matrigel (356230, Corning, Inc., Corning, NY, USA) was thawed overnight at 4 °C. A 96-well plate and pipette tips were pre-cooled. Fifty μL of Matrigel was added to each well and allowed to solidify at 37 °C for 30 min. HUVECs were seeded on the Matrigel at a density of 2 × 10^4^ cells per well in 100 μL of medium and treated with matched concentrations of control (PBS), free anti-VEGFA (10 μg/mL, ab46154, Abcam plc, Cambridge, UK), Rabexo (2 × 10^9^ particles/mL, matching the exosome concentration in the aV-Rabexo group), or aV-Rabexo (antibody concentration 10 μg/mL; exosome concentration 2 × 10^9^ particles/mL). After 6 h of incubation at 37 °C, tubular network formation was photographed using an inverted microscope (DMi8, Leica Microsystems GmbH, Wetzlar, Germany) at 100× magnification. The total branching length and junction number of tube structures were quantitatively analyzed using ImageJ software (version 1.53t, National Institutes of Health, Bethesda, MD, USA) with the Angiogenesis Analyzer plugin. All experiments were performed in triplicate.

### 4.11. Animal Model Establishment and Treatment Protocol

Eight-week-old male BALB/c mice (weighing 20–25 g) were obtained from the Laboratory Animal Center of Jiangnan University (Wuxi, China). All animal procedures were approved by the Institutional Animal Care and Use Committee of Jiangnan University (approval No. 20240915b0640101) and conducted in accordance with the ARVO Statement for the Use of Animals in Ophthalmic and Vision Research. Mice were anesthetized by intraperitoneal injection of 0.3% pentobarbital sodium (40 mg/kg, Sigma-Aldrich, Inc., St. Louis, MO, USA). The right eye was treated with a 2 mm filter paper soaked in 1 M sodium hydroxide (NaOH, Sinopharm Chemical Reagent Co., Ltd., Shanghai, China) for 30 s, followed by immediate 0.9% sterile saline rinse for 1 min. Mice were then treated with levofloxacin eye drops (Cravit, Santen Pharmaceutical Co., Ltd., Noto Plant, Ishikawa, Japan) twice daily for three days to prevent infection. Mice were randomly divided into six groups (*n* = 6 per group): normal control, alkali burn, blank gel, Rabexo-Gel, free antibody gel and aV-Rabexo-Gel. Topical administration (50 μL/eye) was performed on days 0, 4, 7 and 10 after modeling using a micropipette. The treatment groups were designed with matched concentrations: the free anti-VEGFA antibody gel group and the aV-Rabexo-Gel group both contained 10 μg/mL of anti-VEGFA antibody (equivalent to 0.5 μg per eye); the blank Rabexo-Gel group and the aV-Rabexo-Gel group both contained 2 × 10^9^ particles/mL of Rabexo (equivalent to 1 × 10^8^ particles per eye). The gel was allowed to spread evenly over the corneal surface by gently closing the eyelid for 5–10 s after administration.

### 4.12. Histological Staining

At day 14 post-modeling, mouse eyeballs were harvested and fixed in 4% paraformaldehyde overnight at 4 °C. After fixation, tissues were dehydrated through graded ethanol series (70%, 80%, 95%, 100%), cleared in xylene, and embedded in paraffin. Corneal tissue sections (4-μm thickness) were cut using a microtome (RM2235, Leica Biosystems Nussloch GmbH, Nussloch, Germany), mounted on glass slides, deparaffinized in xylene, and rehydrated through graded ethanol. Masson‘s trichrome staining was performed according to the manufacturer’s instructions (G1340, Beijing Solarbio Science & Technology Co., Ltd., Beijing, China) to observe corneal collagen fiber arrangement and structural integrity. Stained sections were observed under a light microscope (DM1000, Leica Microsystems GmbH, Wetzlar, Germany) at 200× magnification. Collagen disorganization was semi-quantitatively evaluated by two independent blinded observers according to a standardized scoring system (0 = normal, 1 = mild, 2 = moderate, 3 = severe).

### 4.13. Immunofluorescence Staining

Frozen corneal sections (8–10 μm thickness, prepared as described in Section 4.7) were fixed with 4% paraformaldehyde for 15 min, permeabilized with 0.2% Triton X-100 (T8200, Beijing Solarbio Science & Technology Co., Ltd., Beijing, China) for 10 min, and blocked with 5% bovine serum albumin (BSA, Sinopharm Chemical Reagent Co., Ltd., Shanghai, China) in PBS for 1 h at room temperature. Sections were incubated with primary antibodies against CD31 (ab28364, 1:200 dilution, Abcam plc, Cambridge, UK) and VEGF (ab46154, 1:100 dilution, Abcam plc, Cambridge, UK) overnight at 4 °C in a humidified chamber. After three PBS washes (5 min each), sections were incubated with corresponding fluorescent secondary antibodies (Alexa Fluor™ 488-conjugated goat anti-rabbit IgG, A-11008, 1:500 dilution; Alexa Fluor™ 594-conjugated goat anti-rabbit IgG, A-11012, 1:500 dilution, Thermo Fisher Scientific, Inc., Waltham, MA, USA) for 1 h at room temperature in the dark. Nuclei were stained with DAPI (62248, 1 μg/mL, Thermo Fisher Scientific, Inc., Waltham, MA, USA) for 5 min. Fluorescence images were captured using a confocal laser scanning microscope (LSM 880, Carl Zeiss AG, Oberkochen, Germany) with identical acquisition settings for all groups. The positive area percentages of CD31 and VEGF were calculated using ImageJ software (version 1.53t, National Institutes of Health, Bethesda, MD, USA).

### 4.14. Western Blot Analysis

Total protein was extracted from corneal tissues using RIPA lysis buffer (P0013B, Beyotime Biotechnology, Shanghai, China) containing protease inhibitor cocktail (P1005, Beyotime Biotechnology, Shanghai, China) and 1 mM PMSF (ST506, Beyotime Biotechnology, Shanghai, China). Tissues were homogenized on ice for 30 s using a tissue grinder, followed by lysis on ice for 30 min with vortexing every 10 min. After centrifugation at 12,000× *g* for 15 min at 4 °C, the supernatant was collected. Protein concentration was determined using a BCA protein assay kit (P0010, Beyotime Biotechnology, Shanghai, China) with BSA as the standard. Equal amounts of protein (30 μg per lane) were separated by 12% SDS-PAGE (P0012A, Beyotime Biotechnology, Shanghai, China) and transferred to a single PVDF membrane (IPVH00010, MilliporeSigma, Burlington, MA, USA) using a wet transfer system (Mini Trans-Blot, Bio-Rad Laboratories, Inc., Hercules, CA, USA) at 300 mA for 90 min on ice. After transfer, the membrane was blocked with 5% non-fat milk (232100, BD Biosciences, Franklin Lakes, NJ, USA) in TBST (Tris-buffered saline with 0.1% Tween-20) for 1 h at room temperature. The membrane was then incubated with primary antibodies against α-SMA (ab32575, 1:1000 dilution, Abcam plc, Cambridge, UK) and β-actin (ab8226, 1:5000 dilution, Abcam plc, Cambridge, UK) overnight at 4 °C. After three TBST washes (10 min each), the membrane was incubated with HRP-conjugated goat anti-rabbit secondary antibody (ab6721, 1:5000 dilution, Abcam plc, Cambridge, UK) for 1 h at room temperature. All electrophoresis, transfer, exposure and imaging parameters remained identical across all rounds of detection. Protein bands were visualized using an enhanced chemiluminescence system (Tanon 5200, Tanon Science & Technology Co., Ltd., Shanghai, China). Relative protein expression levels were quantified by densitometry using ImageJ software (version 1.53t, National Institutes of Health, Bethesda, MD, USA) and normalized to β-actin. All Western blot quantifications were performed by observers blinded to group allocation.

### 4.15. Statistical Analysis

All data were tested for normality using the Shapiro–Wilk test and for homogeneity of variance using Levene’s test. When these assumptions were met, one-way ANOVA with Tukey’s post hoc test was used for multiple group comparisons, and independent samples *t*-test was used for two-group comparisons. When the assumptions were violated, the non-parametric Kruskal–Wallis test (for multiple groups) or Mann–Whitney U test (for two groups) was applied instead. All experimental data were presented as mean ± standard deviation (SD). A value of *p* < 0.05 was considered statistically significant. All experiments were independently repeated at least three times. Statistical analysis was performed using GraphPad Prism (version 10.1.2, GraphPad Software, Inc., San Diego, CA, USA). All Western blot and immunofluorescence quantifications were performed by observers blinded to group allocation.

## 5. Study Limitations and Future Perspectives

Despite the promising therapeutic potential demonstrated, several limitations warrant discussion. First, regarding the mechanism of action, while we confirmed the dual anti-angiogenic and anti-fibrotic effects of aV-Rabexo-Gel, the specific transcellular transport pathways across the corneal epithelium and the precise endocytic mechanisms of Rabexo internalization remain to be fully elucidated. Second, methodological challenges inherent to protein-loaded exosomes persist; specifically, distinguishing encapsulated antibodies from surface-adsorbed ones and characterizing intracellular release kinetics (e.g., endosomal escape) require advanced techniques like surface protease digestion and subcellular fractionation in future studies. Third, the drug loading capacity and hydrogel rheology require further optimization. Additionally, while in vivo data confirmed sustained bioavailability, quantitative release profiles under simulated tear fluid conditions were not established. Fourth, safety assessments were limited to short-term observations. Comprehensive long-term toxicity studies, particularly regarding retinal function and systemic safety in larger animal models, are necessary to validate translational potential. Finally, as a plant-derived preparation, batch-to-batch variability and the potential presence of non-vesicular contaminants necessitate rigorous orthogonal purification and characterization (e.g., cryo-TEM, lipidomics) to ensure consistency.

## Data Availability

The original contributions presented in this study are included in the article. Further inquiries can be directed to the corresponding author.

## References

[1] Chang J.-H., Gabison E.E., Kato T., Azar D.T. (2001). Corneal Neovascularization. Curr. Opin. Ophthalmol..

[2] Zeng Z., Li S., Ye X., Wang Y., Wang Q., Chen Z., Wang Z., Zhang J., Wang Q., Chen L. (2024). Genome Editing *VEGFA* Prevents Corneal Neovascularization In Vivo. Adv. Sci..

[3] Wilson S.E. (2020). Corneal Myofibroblasts and Fibrosis. Exp. Eye Res..

[4] Chang C.-H., Peng I.-C., Huang Y.-H. (2025). Recombinant Thrombomodulin Domain 1 Modulates Macrophage Polarization and Enhances Healing in Corneal Alkali Burns. Investig. Ophthalmol. Vis. Sci..

[5] Stevenson W., Cheng S.-F., Dastjerdi M.H., Ferrari G., Dana R. (2012). Corneal Neovascularization and the Utility of Topical VEGF Inhibition: Ranibizumab (Lucentis) vs Bevacizumab (Avastin). Ocul. Surf..

[6] Lyu N., Zhao Y., Xiang J., Fan X., Huang C., Sun X., Xu J., Xu Z.P., Sun J. (2021). Inhibiting Corneal Neovascularization by Sustainably Releasing Anti-VEGF and Anti-Inflammation Drugs from Silica-Thermogel Nanohybrids. Mater. Sci. Eng. C.

[7] Huang X., Xu G., Yang P., Wang L., Wang H., Hu Y. (2025). Recent Progress on Topical Drug Therapy for Corneal Neovascularization. ChemMedChem.

[8] Wu D., Chan K.E., Lim B.X.H., Lim D.K.-A., Wong W.M., Chai C., Manotosh R., Lim C.H.L. (2024). Management of Corneal Neovascularization: Current and Emerging Therapeutic Approaches. Indian J. Ophthalmol..

[9] Soleimani M., Mirshahi R., Cheraqpour K., Baharnoori S.M., Massoumi H., Chow C., Shahjahan S., Momenaei B., Ashraf M.J., Koganti R. (2023). Intrastromal versus Subconjunctival Injection of Mesenchymal Stem/Stromal Cells for Promoting Corneal Repair. Ocul. Surf..

[10] Sun C., Ruan F., Li S., Zhang J.-Q., Jie Y. (2023). Subconjunctival Conbercept for the Treatment of Corneal Neovascularization. Int. J. Ophthalmol..

[11] Sadeghi S., Tehrani F.R., Tahmasebi S., Shafiee A., Hashemi S.M. (2023). Exosome Engineering in Cell Therapy and Drug Delivery. Inflammopharmacology.

[12] Serrano D.R., Juste F., Anaya B.J., Ramirez B.I., Sánchez-Guirales S.A., Quispillo J.M., Hernandez E.M., Simon J.A., Trallero J.M., Serrano C. (2025). Exosome-Based Drug Delivery: A Next-Generation Platform for Cancer, Infection, Neurological and Immunological Diseases, Gene Therapy and Regenerative Medicine. Pharmaceutics.

[13] Tian Y., Zhang F., Qiu Y., Wang S., Li F., Zhao J., Pan C., Tao Y., Yu D., Wei W. (2021). Reduction of Choroidal Neovascularization via Cleavable VEGF Antibodies Conjugated to Exosomes Derived from Regulatory T Cells. Nat. Biomed. Eng..

[14] Zhou T., He C., Lai P., Yang Z., Liu Y., Xu H., Lin X., Ni B., Ju R., Yi W. (2022). miR-204–Containing Exosomes Ameliorate GVHD-Associated Dry Eye Disease. Sci. Adv..

[15] Tian Y., Zhang T., Li J., Tao Y. (2023). Advances in Development of Exosomes for Ophthalmic Therapeutics. Adv. Drug Deliv. Rev..

[16] Zheng B., Wang X., Guo M., Tzeng C.-M. (2025). Current Development of Mesenchymal Stem Cell-Derived Extracellular Vesicles. Cell Transpl..

[17] Castiglione K., Fuhrmann G. (2026). Overcoming the Barriers to the Scaled-up Production of Therapeutic Bacterial Extracellular Vesicles. Nanomedicine.

[18] Tinnirello V., Rabienezhad Ganji N., De Marcos Lousa C., Alessandro R., Raimondo S. (2023). Exploiting the Opportunity to Use Plant-Derived Nanoparticles as Delivery Vehicles. Plants.

[19] Jin E., Yang Y., Cong S., Chen D., Chen R., Zhang J., Hu Y., Chen W. (2025). Lemon-Derived Nanoparticle-Functionalized Hydrogels Regulate Macrophage Reprogramming to Promote Diabetic Wound Healing. J. Nanobiotechnol..

[20] Bushra A., Fall M., Ren W., Igbalaye J., Irudayaraj J. (2025). Plant-Derived Exosome-Coated Niosome Oxygen Nanobubbles for the Mitigation of Ocular Ischemia. ACS Pharmacol. Transl. Sci..

[21] Yang C., Zhang M., Merlin D. (2018). Advances in Plant-Derived Edible Nanoparticle-Based Lipid Nano-Drug Delivery Systems as Therapeutic Nanomedicines. J. Mater. Chem. B.

[22] Weng J., Chen Y., Zeng Y., Jin W., Ji Y., Zhang W., Wang S., Li H., Yi M., Niu X. (2025). A Novel Hydrogel Loaded with Plant Exosomes and Stem Cell Exosomes as a New Strategy for Treating Diabetic Wounds. Mater. Today Bio.

[23] Kang S.J., Lee J.H., Rhee W.J. (2024). Engineered Plant-Derived Extracellular Vesicles for Targeted Regulation and Treatment of Colitis-Associated Inflammation. Theranostics.

[24] Wang T., Li Y., Hao L., Liu Y., Liu D., Zhang C., Yi H., Zhang J. (2025). Coriander-Derived Exosome-Like Nanovesicles Laden Hydrogel with Antioxidant Property Accelerates Wound Healing. Macromol. Biosci..

[25] Sabin O., Pop R.M., Bocșan I.C., Chedea V.S., Ranga F., Grozav A., Levai A.-M., Buzoianu A.D. (2024). The Anti-Inflammatory, Analgesic, and Antioxidant Effects of Polyphenols from Brassica Oleracea Var. Capitata Extract on Induced Inflammation in Rodents. Molecules.

[26] Cao J., Zhang J., Yan L., Liu C., Wang Y., Cao Y., Zheng L. (2025). Red Cabbage Exosome-like Nanoparticles Inhibited LPS-Induced Inflammation and Oxidative Stress in RAW264.7 Cells. Food Sci. Biotechnol..

[27] Lu X.-X., Zhao S.-Z. (2019). Gene-Based Therapeutic Tools in the Treatment of Cornea Disease. Curr. Gene Ther..

[28] Zhao B., Lin H., Jiang X., Li W., Gao Y., Li M., Yu Y., Chen N., Gao J. (2024). Exosome-like Nanoparticles Derived from Fruits, Vegetables, and Herbs: Innovative Strategies of Therapeutic and Drug Delivery. Theranostics.

[29] Bai C., Liu J., Zhang X., Li Y., Qin Q., Song H., Yuan C., Huang Z. (2024). Research Status and Challenges of Plant-Derived Exosome-like Nanoparticles. Biomed. Pharmacother..

[30] Lian M.Q., Chng W.H., Liang J., Yeo H.Q., Lee C.K., Belaid M., Tollemeto M., Wacker M.G., Czarny B., Pastorin G. (2022). Plant-derived Extracellular Vesicles: Recent Advancements and Current Challenges on Their Use for Biomedical Applications. J. Extracell. Vesicle.

[31] You J.Y., Kang S.J., Rhee W.J. (2021). Isolation of Cabbage Exosome-like Nanovesicles and Investigation of Their Biological Activities in Human Cells. Bioact. Mater..

[32] Liu X., Wang X., Li J., Zhao Z., Liu Y., Li X., Wang W., Wang Q., Sun X., Guo M. (2025). Cabbage Exosome-Like Nanoparticles Encapsulating Small Noncoding tsRNA Prevent Postinjury Arterial Restenosis. Research.

[33] Li R., Chen Y., Zhou Y., Zhang L., Luo X., Guan A., Zhou A., Yao T., Yang C., Li K. (2025). PVA/HA@BT Hydrogel with Antioxidant and Antibacterial Abilities for Accelerating Diabetic Wound Healing. Int. J. Biol. Macromol..

[34] Sabbagh F., Zargarian S.S., Kosik-Kozioł A., Nakielski P., Pierini F. (2025). Hydrogel-Based Ocular Drug Delivery Systems. J. Mater. Chem. B.

[35] Zhu W., Xia M., He Y., Huang Q., Liao Z., Wang X., Zhou X., Duan X. (2026). Hydrogels as Promising Carriers for Ophthalmic Disease Treatment: A Comprehensive Review. Gels.

[36] Ahmad A., Nawaz M.I. (2022). Molecular Mechanism of VEGF and Its Role in Pathological Angiogenesis. J. Cell. Biochem..

[37] Xie M., Jie Y. (2025). Transforming Corneal Alkali Burn Treatment: Unveiling Mechanisms and Pioneering Therapies from Bench to Bedside. J. Transl. Med..

[38] Wang C., Yuan K., Wu Y., Jiang T., Mou Y., Wang N., Chen X., Yu J., Yang S., Jin X. (2025). Targeting ER Stress/ROS-Driven VEGF Signaling Axis: AdipoRon as a Multifunctional Therapeutic Agent for Alkali Burn-Induced Corneal Neovascularization. Free Radic. Biol. Med..

